# Does Thoracic Manipulation Cause Extravasation at Joint Following Facet Injections?

**DOI:** 10.7759/cureus.11340

**Published:** 2020-11-05

**Authors:** Ryan C McCoy, William Clifton, Joseph M Accurso, Mark Friedrich Hurdle

**Affiliations:** 1 Physical Therapy, Mayo Clinic, Jacksonville, USA; 2 Neurological Surgery, Mayo Clinic, Jacksonville, USA; 3 Radiology, Mayo Clinic, Jacksonville, USA; 4 Pain Management, Mayo Clinic, Jacksonville, USA

**Keywords:** thoracic manipulation, facet injection, pain management, ultrasound guided injection

## Abstract

Facet injections and other pain management interventions are commonly performed in combination with conservative therapy to address spinal pain. Joint mobilizations are a highly utilized intervention for manual practitioners to treat patients with spinal pain. Clinical reasoning and decision making models have not been well described in the literature assessing if and when joint mobilizations are appropriate interventions immediately or shortly following facet injection procedures. It has not been well studied if joint mobilizations immediately following facet injections negatively impact the injected solution at the respective joint and thus influence therapeutic effect. More specifically, there is a paucity of evidence assessing this at the thoracic spine. The purpose of this study was to assess if thoracic joint high-velocity low amplitude thrust manipulations caused extravasation of injected radiolucent material at respective thoracic facet joints on a cadaver. This study included an expert physician performing ultrasound-guided facet injections, an experienced manual physical therapist performing joint mobilization techniques, and fluoroscopic assessment of radiolucent material pre- and post-manipulation by a board-certified radiologist with experience in this field of study. Imaging interpretation confirmed that extravasation at respective joints did not occur following manipulation. This basic research can help guide clinical reasoning for practitioners considering implementing manual therapy techniques following facet injections and help guide further research.

## Introduction

Back pain is a common cause for an individual to seek help from healthcare providers. Back pain is commonly treated in physical therapy and concurrently by pain management providers for chronic conditions in multi-disciplinary based healthcare systems. Thoracic back pain, though not as common as lumbar or cervical pain, is present in approximately 15% of adults [1]. The prevalence of mid-back and upper back pain secondary to involvement of the facet joints has been reported in controlled studies in as many as 34% to 48% of patients [2-6]. Ultrasound-guided facet injections are a common procedure for treating facet pathology of the thoracic spine [7-9]. Ultrasound-guided thoracic facet injection procedures have been well described in the literature and are commonly utilized in clinical practice [8,9]. In addition, manual therapy techniques including graded mobilizations and joint manipulation are commonly utilized by physical therapists, chiropractors, and osteopathic health care providers as a common intervention for treating facetogenic based thoracic back pain [10-12]. Varying treatment rationales exist in physical therapy immediately following facet injections such as whether to utilize high-velocity low amplitude thrust techniques days following the procedure. This in part can be due to patient presentation, referring provider guidelines based on clinical expertise, tissue healing time frames, or treating therapist preference to avoid interfering with the potential therapeutic benefits at the recently injected joint. It is common for manual practitioners to avoid or delay application of high-velocity low amplitude joint manipulation maneuvers shortly after facet injections to potentially avoid altering or interfering with the procedures therapeutic effect. Few studies have looked at the efficacy of combining interventional facet injections and manual therapy techniques to help manage pain and dysfunction [13-15]. Outcomes from these studies assessed therapeutic effect on pain and function when combining the interventions rather than assessing if one intervention or variable impacts the other. It has not been well studied if extravasation of injected solution occurs at the facet joint when a joint manipulation technique is utilized immediately following the injection procedure. Due to the paucity of evidence in this area, there is a gap in clinical understanding and further assessment of this can improve clinical reasoning and guide future treatments if deemed appropriate by the treating providers and patient. The purpose of this study was to assess if thoracic joint manipulation causes extravasation of solution at the respective facet joint immediately following thoracic facet joint injections.

## Materials and methods

This study began by selecting a full body cadaver that was completely thawed to facilitate normal tissue properties and was placed prone on an adjustable Skytron surgical table (Skytron LLC, Grand Rapids, MI, United States) with arms by each side. An experienced pain medicine physician mapped specific thoracic segments via C-Arm fluoroscopy on the cadaver. Ultrasound guidance was utilized by the expert pain medicine physician to visualize and inject the intended thoracic facet joints. C-Arm fluoroscopy was utilized to visualize the injected solution into each intended facet. A 22G spinal needle that was 3.5 inches long and a 10ml syringe was utilized for the injection. The dye was a fluoroscopy solution called Omnipaque (GE Healthcare, Lithia Springs, GA, USA). Bilateral seventh and eighth thoracic vertebrae (T7-8) facets were injected individually and visualized both during and after injection which was taken via fluoroscopy. Immediately following the injection and visualization of radiolucent dye at the respective facet joints, three consecutive trials of a posterior to anterior high-velocity low amplitude thrust wind up technique was performed by an experienced manual physical therapist at that same T7-8 spinal segment. The wind-up technique was applied with the practitioner standing at the left side of the table with placement of the right hypothenar eminence on the left transverse process one level inferior to intended segment and the left thenar eminence on the right transverse process one level superior to the intended segment. Soft tissue slack was taken up while translating point of contact through right hypothenar superiorly and the left thenar eminence inferiorly to ipsilateral transverse process of intended segment. Practitioner position can be altered to either side of the table if mobilization preference exists. Imaging was then taken again at the same spinal segment to assess if extravasation at the respective facet joints occurred. The same sequence was then performed at the right eighth and ninth thoracic facet joint (right T8-9) and then at the left ninth and tenth thoracic facet joint (left T9-10). Images were reviewed by a board-certified radiologist with experience in image-guided joint injections.

## Results

**Figure 1:**
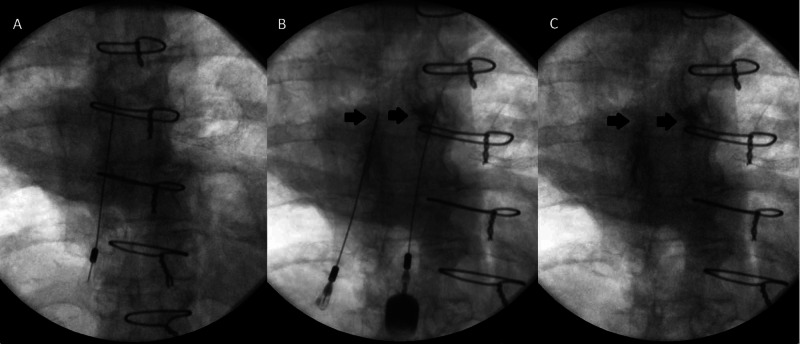
Fluoroscopic imaging of facet joint injection at seventh and eighth thoracic facet joint (T7/8) pre and post high velocity low amplitude thrust manipulation. (A) Visualization of needle at left seven and eighth thoracic facet joint (T7/8) prior to injection. (B) Bilateral thoracic facet joint injection with dense radiolucent dye visualized at insertion of each needle with sharp margins. (C) No extravasation of contrast from the joint space is visualized following three trials of manipulation.

**Figure 2:**
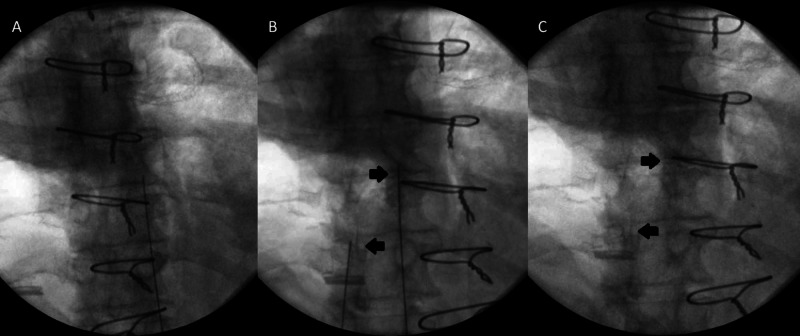
Fluoroscopic image of unilateral thoracic facet joint injection with radiolucent dye pre and post high velocity low amplitude thrust manipulations. A. Visualization of needle prior to injection at right thoracic eighth and ninth facet segment. B. Arrows highlighting dense radiolucent dye solution injected at respective unilateral thoracic facet joints with sharp margins. C. No extravasation of contrast from the joint space is visualized following segmental manipulations. Appreciation of proximal bilateral facet joint injection also visualized and remains unchanged following regional thoracic high velocity low amplitude thrust manipulations.

The Omnipaque dye solution was injected at varying thoracic facet joints and remained at each respective facet joint following three trials of high-velocity low amplitude thrust manipulations at each segment (Figure 1, 2). The bilateral seventh and eighth thoracic facet joint (T7-8), the right eighth and ninth facet joint (right T8-9), and lastly the left ninth and tenth thoracic facet joint (left T9-10) were injected and successfully mobilized. 

## Discussion

The images demonstrated accurate placement of the injection needle(s) in the targeted facet joints, with deposition of radiopaque contrast into the joint space. Images obtained after injection but prior to the facet joint manipulation demonstrate dense contrast with sharp margins. Post manipulation there is reduced contrast density and contrast more diffusely distributed throughout the joint. No extravasation of contrast from the joint space is visualized. The results of this study confirmed extravasation of the solution did not occur at the respective facet joints when local and regional wind up manipulative joint techniques were applied after ultrasound guided facet injections. Commonly, spinal manipulation or mobilization techniques are avoided in areas by manual practitioners immediately or shortly following facet joint injections with fear of negatively impacting the therapeutic effect. Results from this study indicate that applying the described manual techniques after an ultrasound guided facet injection does not negatively impact the therapeutic effect of the injection procedure with respect to extravasation of the solution in a cadaveric model. This provides appropriate clinicians insight for clinical reasoning strategies such as implementation of described thoracic manual therapy techniques following ultrasound guided facet joint injection for a combined treatment approach if deemed appropriate. This can also help guide future research for a combined therapy approach for additional pain management strategies.

Limitations of this basic research study include utilization of a single cadaver. Also, this cadaveric model may differ from an in vivo study with understood differences in tissue properties, capillary blood flow at the local facet joint, and patient tolerance of manipulation techniques immediately following facet joint injection. Further limitations include visualization of the facet joint space is somewhat limited on single projection C-Arm radiograph whereas CT scanning of pre and post manipulation facet joints would significantly improve visualization of contrast including more accurate evaluation for extravasation from the joint space. Though limitations exist, this research model can help guide clinicians and future studies assessing combined pain management intervention strategies. 

## Conclusions

The aim of this study was to assess if high-velocity low amplitude thrust techniques caused extravasation at the facet joint following respective joint injection in order to enhance clinical reasoning strategies for healthcare providers utilizing or considering this pain management strategy. Based on this cadaveric model, extravasation from the facet joints did not occur with described manipulative techniques immediately following facet injections. 
